# Necrotizing Soft Tissue Infection of the Breast: A Unique Presentation of Underlying Invasive Breast Cancer

**DOI:** 10.7759/cureus.87310

**Published:** 2025-07-05

**Authors:** Oscar Rios Herrera, Maria De La Torre, Siarrhei Melnikau, Nischal Bogati, Kaleab Debebe, Antonio Melhem, Rollin William Johnson, Jessica Pagé, Aleksandra Zamaro, Tereque Raeburn

**Affiliations:** 1 Surgery, Wyckoff Heights Medical Center, Brooklyn, USA; 2 General Surgery, Wyckoff Heights Medical Center, Brooklyn, USA; 3 Breast Surgery, Wyckoff Heights Medical Center, Brooklyn, USA; 4 General Surgery, St. George's University School of Medicine, St. George's, GRD

**Keywords:** breast cancer, critical care, invasive ductal breast carcinoma, necrotizing soft tissue infection, sepsis, surgical oncology

## Abstract

Necrotizing soft tissue infections (NSTIs) are life-threatening infections that most commonly affect the extremities, perineum, and abdominal wall. These infections begin with the presence of toxin-producing bacteria that invade through a defect in the skin barrier, such as a wound, laceration, trauma, or recent surgical incision. These bacteria cause subsequent tissue destruction and necrosis that can involve the superficial skin, subcutaneous tissue, fascia, and/or muscle. NSTIs can progress quickly, leading to severe sepsis, shock, and even death. NSTIs associated with the breast are an exceedingly rare occurrence, requiring early diagnosis and prompt surgical intervention. In this article, we report the case of a 46-year-old woman with an NSTI of the left breast, which required serial debridement initially, and subsequently a modified radical mastectomy given a pathological diagnosis of invasive ductal carcinoma.

## Introduction

Necrotizing soft tissue infections (NSTIs) are severe infections characterized by rapidly progressive necrosis and can involve all layers of soft tissue, superficial and/or deep. The extremities are the most common site of infection (58%), followed by the abdominal wall and perineum [1]. Early source control through surgical intervention, broad-spectrum antimicrobial therapy, and critical supportive measures are the cornerstones of effective management. The urgency of surgical source control is particularly high when clinical suspicion is significant, as delayed treatment correlates with worse outcomes. Necrotizing infections of the breast are exceedingly rare and present a unique clinical challenge. There are less than 50 published case reports and case series of NSTIs of the breast, and an added rarity of this case is the likelihood of the breast cancer inciting the necrotizing infection [2]. The following case outlines a unique presentation of a human epidermal growth factor receptor 2 (HER2)-positive breast cancer, in the setting of a necrotizing infection of the breast.

## Case presentation

Presentation

**Figure 1:**
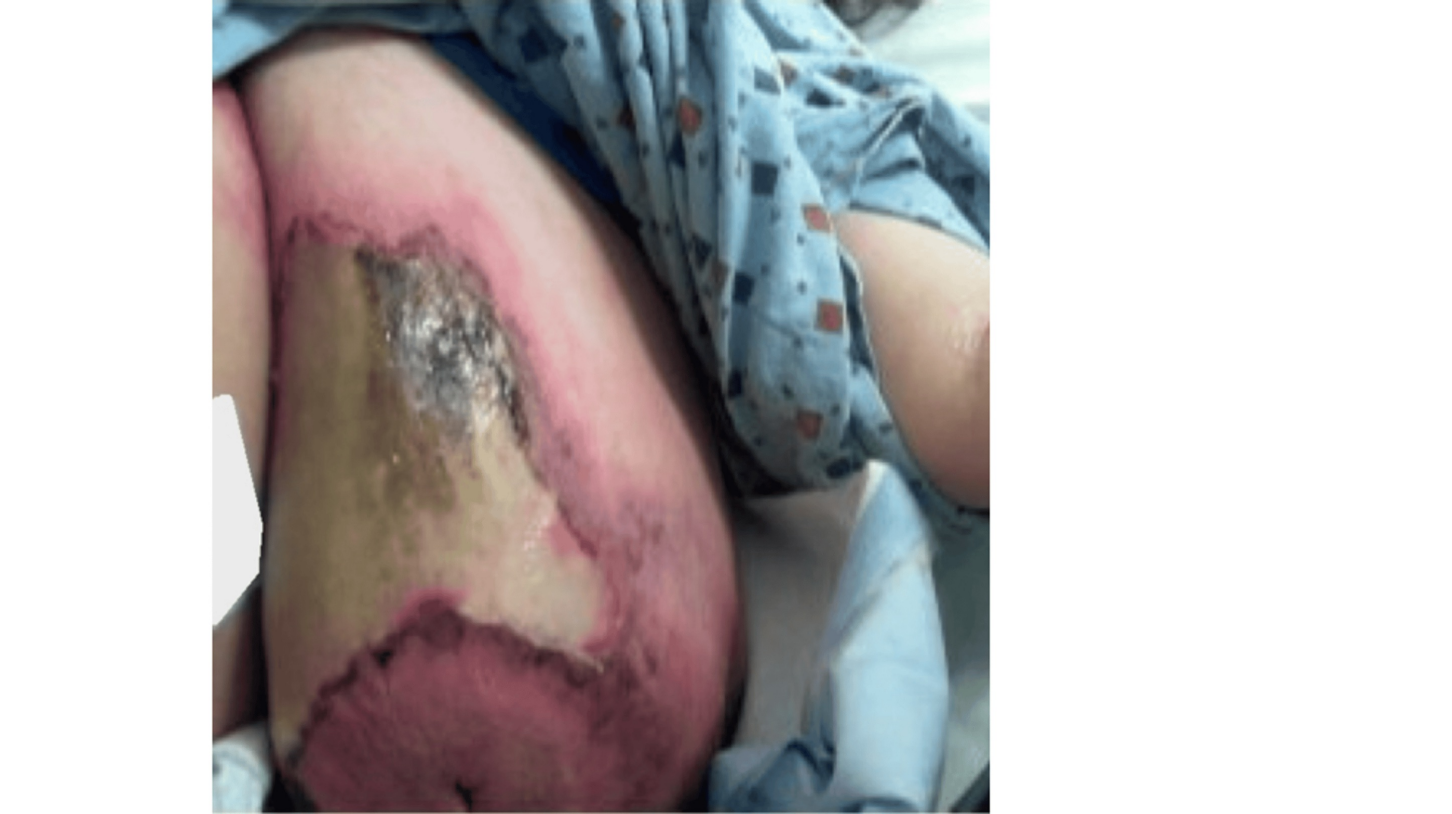
Presentation of the left breast

A 46-year-old woman presented to the emergency department (ED) in late February 2024 with a three-day history of erythema, warmth, pain, skin sloughing, and foul-smelling sanguinopurulent discharge localized to the inframammary region of her left breast. She denied any recent trauma, local wounds, fever, chills, weight loss, or chronic fatigue. Her medical history was notable for trichorhinophalangeal syndrome, sciatica, vertigo, and morbid obesity (BMI 49.5). Surgical history was noncontributory. Figure 1 shows the presentation of the left breast.

Physical examination

Initial vital signs revealed a heart rate of 137 bpm, blood pressure of 120/100 mmHg, respiratory rate of 18-22 breaths per minute, and oxygen saturation of 97% on room air. The patient had altered mental status and developed respiratory distress; she was then transitioned to noninvasive positive pressure ventilation. On examination, the left breast was noted to be swollen with periareolar erythema, mild retraction, and sloughing of the skin with ischemic tissue changes. A firm, 6 x 6 cm mass was palpated in the upper outer quadrant and deep to the chest wall, and axillary exam revealed lymph nodes that were matted. No crepitus or nipple discharge was appreciated. The right breast was unremarkable.

Initial labs, imaging, and ICU admission

**Figure 2:**
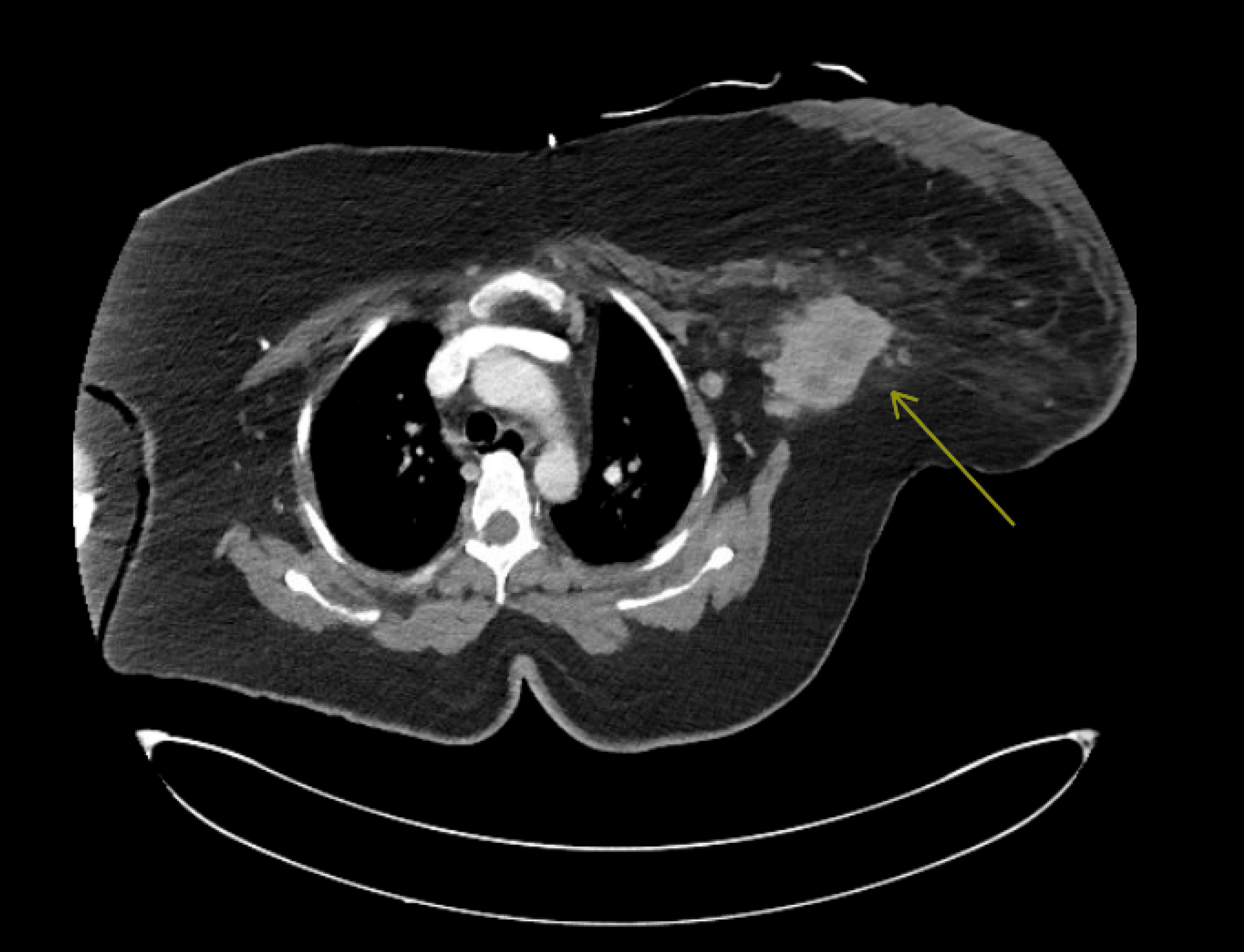
Index CT showing deep mass, which is partially covered by the pectoralis major muscle. Adjacent pathologic nodes just lateral to the pectoralis minor muscle are visible. The mass itself measured radiographically close to 5.3 x 5.3 cm CT: computed tomography

Initial labs revealed significant lactic acidosis, acute kidney injury, and leukocytosis (Table 1). The Laboratory Risk Indicator for Necrotizing Fasciitis (LRINEC) score was at a 6, placing the patient at intermediate risk. Two large-bore peripheral lines were inserted for the patient, and she received a bolus of 3 L of intravenous fluids, to which she responded. Empiric broad-spectrum antibiotic therapy was started in the ED with vancomycin, piperacillin-tazobactam, and clindamycin. A computed tomography (CT) scan of the chest demonstrated skin thickening over the left breast, a deep-seated mass measuring approximately 5.3 x 5.3 cm partially located beneath the pectoralis major muscle, and pathologically appearing lymph nodes lateral to the pectoralis minor. Figure 2 shows the CT scan and the associated mass. Due to clinical deterioration, including hypotension requiring norepinephrine drip, the patient was emergently transferred to the intensive care unit (ICU) and taken to the operating room for source control.

**Table 1:** Initial lab values

Test	Result	Unit	Reference range
Lactate	5.3	mmol/L	0.5-2.2
Creatinine	3.4	mg/dL	0.6-1.3
Blood urea nitrogen	38	mg/dL	7-20
White blood cells	12.9	k/μL	4-11
Neutrophils	72	%	40-60
Glucose (serum)	138	mg/dL	70-99 (fasting)

Operative findings and clinical course

**Figure 3:**
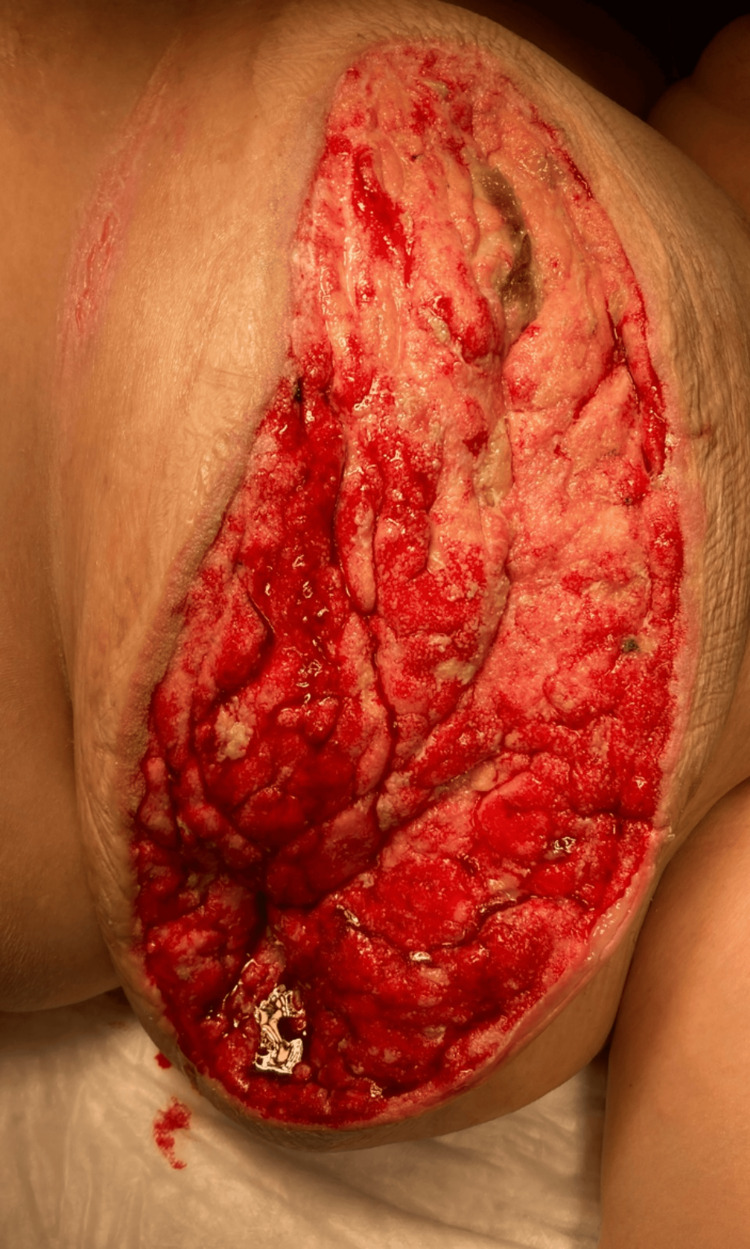
Image before mastectomy, after serial debridements and negative pressure therapy

**Figure 4:**
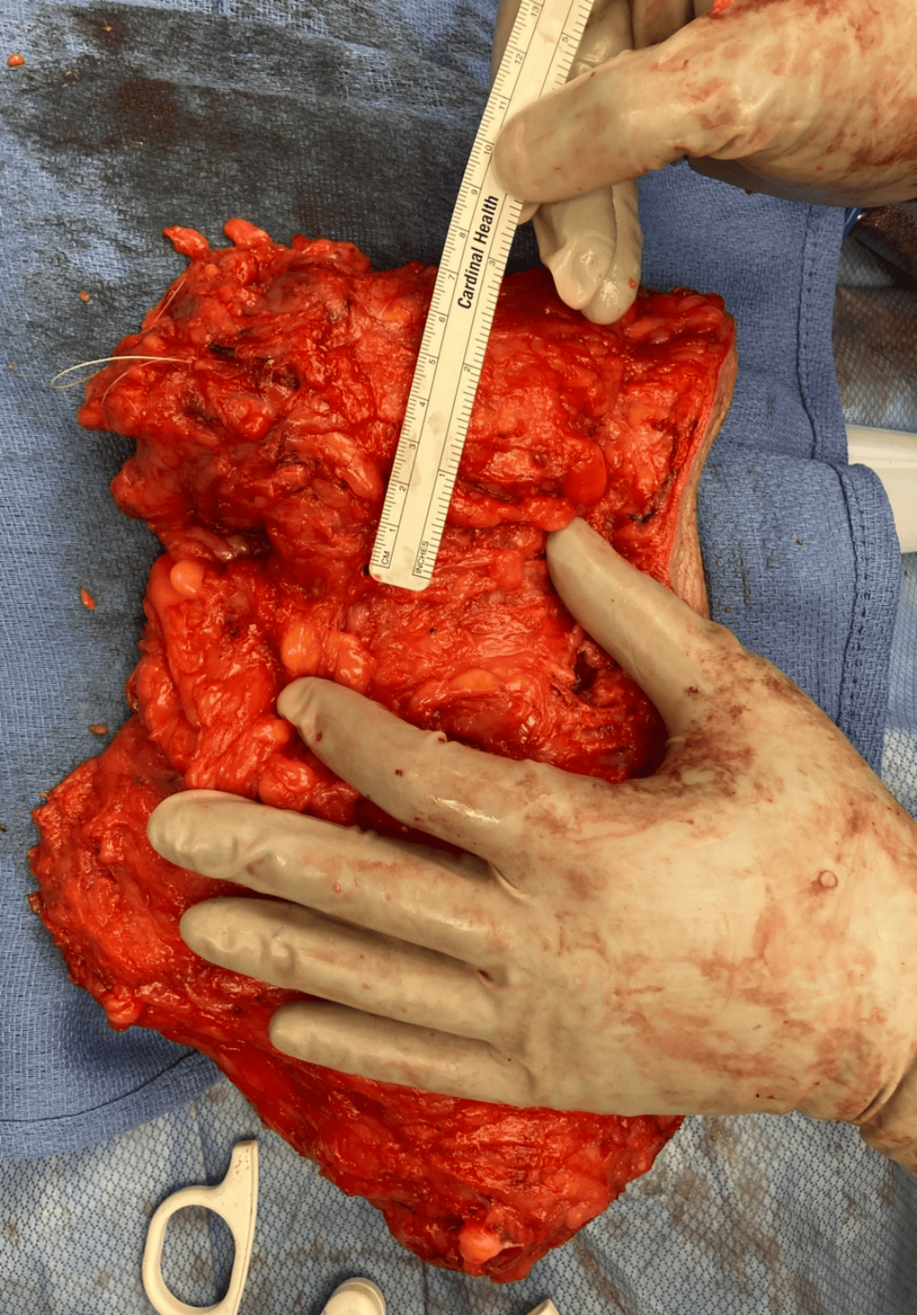
Left mastectomy specimen

Intraoperatively, there was extensive periareolar and breast tissue necrosis with purulent exudate involving a large necrotic mass. Initial surgical management involved debridement of necrotic tissue and incisional biopsy of the mass due to the high suspicion of concomitant breast cancer. The tissue pathology dimensions at the index surgery were 16 cm × 4 cm × 3 cm. The wound was left open with a wet-to-dry dressing for reassessment, and the patient returned to the ICU, intubated and supported by two vasopressors. At the planned second-look surgery after 24 hours, minimal additional necrosis was found, and the remaining breast tissue was viable. Negative pressure wound therapy was placed on the defect at the second-look operation. Notably, the ICU course was complicated by propofol infusion syndrome, which resolved after cessation of that agent and did not worsen the kidney function. She was subsequently extubated and weaned off vasopressors in the subsequent days; the ICU course totaled seven days. Serial debridements were performed, and negative-pressure wound therapy was continued. Pathology results revealed an estrogen receptor-negative, progesterone receptor-negative, HER2-positive invasive moderately differentiated ductal carcinoma, with no component of ductal carcinoma in situ, with lymphovascular invasion and permeation not being identified. Although the standard of care for HER2-positive breast cancer typically involves neoadjuvant chemotherapy, a multidisciplinary team, including breast surgeons, oncologists, and radiation oncologists, elected that performing a definitive surgical oncological resection, a modified radical mastectomy (MRM), in this clinical setting was justified and reasonable. The timeline from the index operation and the definitive MRM was 15 days. The delay of the definitive management was due to the patient's tumultuous hemodynamic status, addressing septicemia, operating room time, and staff availability. The patient tolerated the surgery well and had an uncomplicated postoperative course after the MRM. Figure 3 shows the left breast before the MRM, and Figure 4 shows the final specimen.

## Discussion

NSTIs of the breast can present with nonspecific symptoms and are frequently misdiagnosed as mastitis, abscess, or inflammatory breast cancer, leading to delays in appropriate treatment [1,3]. The unusual presentation in the breast adds further complexity to the timely diagnosis. The rich vascular supply of the breast may contribute to the delayed onset of severe symptoms, resulting in late clinical presentation.

NSTIs are categorized microbiologically into four types: 1) type I: polymicrobial infections, typically seen in patients with underlying comorbidities; 2) type II: monomicrobial infections, commonly caused by *Streptococcus pyogenes* or *Staphylococcus aureus*; 3) type III: infections caused by Clostridium or Vibrio species; and 4) type IV: fungal infections following trauma or burns [4,5].

In this case, blood and wound cultures confirmed monomicrobial *S. pyogene*s, which were pan-sensitive to antibiotics, indicating a Type II infection. Imaging studies, particularly CT, can assist in identifying fascial involvement, edema, and soft tissue gas, although definitive management should not be delayed by imaging [4,5]. The initial CT scan and clinical presentation raised suspicion of an underlying cancer in the setting of an NSTI.

A component of the carcinoma was likely necrotizing, which led to the infectious events. There is a definite gap in the literature documenting rare cases like these and recognition of this pathological process. From the limited available literature, a systematic review by Cai et al. identified two cases where necrotizing fasciitis of the breast led to the diagnosis of previously undiagnosed breast cancer, similar to this case [2].

Pathologically, a malignancy can predispose NSTIs through both local and systemic effects. Locally, tumor invasion, ulceration, or necrosis disrupts normal tissue barriers, providing a portal of entry for bacteria. Systemically, cancer impairs immune function, reducing the host’s ability to contain and clear infections. Once bacteria gain access, the compromised immune response allows rapid proliferation and spread along fascial planes [5].

On the other hand, in the postoperative and trauma setting, there are more published cases in the literature that recognize breast NSTIs. The challenge remains in distinguishing those NSTIs from cellulitis or normal postoperative changes. However, clinical signs including disproportionate pain, skin necrosis, hemorrhagic bullae, and rapid deterioration are key distinguishing features [6-8].

While the LRINEC score has been promoted as a tool to distinguish NSTIs from other infections, its predictive value remains limited in atypical anatomical regions, such as the breast. When calculated for this case, the score was 6 points for C-reactive protein and creatinine elevation.

Prompt surgical exploration remains the gold standard in suspected NSTIs. The more modern way of managing these particular cases now begins with staged debridement rather than immediate mastectomy in breast NSTIs, reserving more definitive surgery until infection is controlled [2,9]. In this case, however, the presence of a confirmed HER2-positive carcinoma necessitated an MRM to achieve both therapeutic goals.

## Conclusions

Necrotizing infections of the breast are rare and pose a significant diagnostic and clinical management challenge. Early recognition, aggressive debridement, and broad-spectrum antibiotics remain the standard of care. When there is a suspicion of an underlying malignancy, multidisciplinary collaboration is essential to achieving optimal outcomes. To date, there is limited literature on NSTI of the breast, particularly in the setting of an undiagnosed breast cancer. This novel case highlights the importance of maintaining a high index of suspicion for NSTI in atypical anatomical locations and adapting management strategies to address concurrent pathologies.
